# Robotic left transthoracic left main bronchial sleeve resection with preoperative three-dimensional planning

**DOI:** 10.1007/s11748-026-02360-1

**Published:** 2026-08-14

**Authors:** David Downes, Benjamin Gerhardy, Jonathan P. Williamson, Benjamin J. H. Ng, Wendy A. Cooper, Christopher Cao

**Affiliations:** 1 School of Medicine, University of New England, NSW Armidale, Australia; 2 Nepean Blue Mountains Local Health District, Nepean Hospital, NSW Kingswood, Australia; 3 Department of Thoracic Medicine, Liverpool Hospital, Sydney, NSW Australia; 4 Respiratory Medicine, MQ Health, Macquarie University Hospital, Sydney, NSW Australia; 5 Tissue Pathology and Diagnostic Oncology, NSW Health Pathology, Royal Prince Alfred Hospital, Camperdown, NSW Australia; 6 Faculty of Health and Medicine, University of Sydney, Sydney, NSW Australia; 7 Department of Cardiothoracic Surgery, Royal Prince Alfred Hospital, The University of Sydney, Camperdown, NSW Australia; 8 Chris O’Brien Lifehouse, Sydney, NSW Australia

**Keywords:** Bronchial sleeve resection, Robotic-assisted thoracic surgery, Left main stem bronchus

## Abstract

Robotic left main bronchial sleeve resection for endobronchial tumors avoids pneumonectomy while preserving the entire ipsilateral lung parenchyma. Preoperative three-dimensional computed tomography reconstruction guides dissection around the pulmonary artery, pulmonary veins, and left atrium, and Firefly fluorescence imaging enables precise intraoperative tumor localization without needle puncture. We hereby describe a patient with a typical carcinoid tumor of the left main bronchus, who successfully underwent a minimally invasive lung -preserving operation to achieve negative surgical margins via a left transthoracic approach.

**Supplementary Information:**

The online version contains supplementary material available at https://doi.org/10.1007/s11748-026-02360-1.

## Introduction

Bronchopulmonary carcinoid tumours are relatively uncommon and are associated with favourable long-term survival following complete surgical resection with negative margins [1]. A subset of patients presents with endobronchial involvement, for whom lung-sparing approaches may be appropriate. In particular, sleeve resections have been associated with significantly lower short-term morbidity and mortality compared with pneumonectomy, as well as improved long-term survival outcomes [2]. Although technically more demanding, sleeve resections can be performed using minimally invasive techniques, including robotic-assisted platforms. Prior studies have demonstrated advantages of the robotic approach over conventional thoracoscopic surgery, including enhanced three-dimensional (3D) visualization and articulated instruments offering seven degrees of freedom of movement [3]. Additionally, patient-specific 3D reconstruction of imaging has emerged as a valuable adjunct for preoperative planning, potentially improving surgical precision and reducing conversion rates [4]. We report a case of robotic-assisted left main bronchial sleeve resection for carcinoid tumour following preoperative 3D planning.

## Case presentation

A 63-year-old retired serviceman presented with a 12-month history of progressive dyspnea, cough, intermittent hemoptysis, and sputum production, accompanied by reduced exercise tolerance. Computed tomography (CT) identified a 13 mm lesion within the left main bronchus. Flexible bronchoscopy confirmed an endobronchial lesion, which was partially debulked, with histopathological analysis reported as a typical carcinoid tumour. A DOTATATE scan demonstrated no evidence of nodal or distant metastatic disease. Following multidisciplinary team discussion, the patient was referred for surgical management. Given the patient’s preference to avoid pneumonectomy, a lung-sparing, minimally invasive robotic left main bronchial sleeve resection was proposed.

**Fig. 1:**
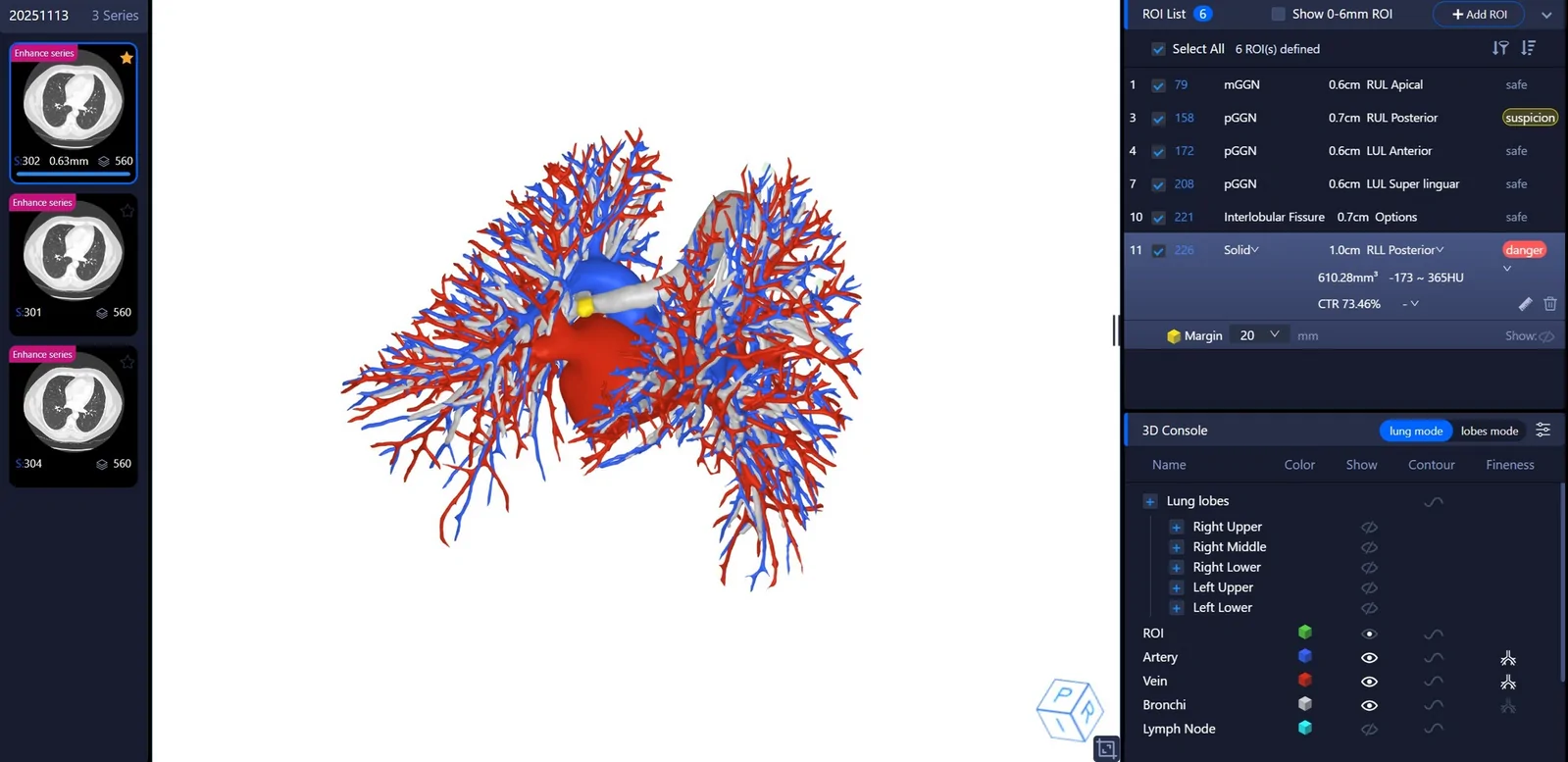
3D image reconstructed in Infervision from the preoperative CT images. The area in yellow indicates tumor location

Preoperative CT imaging was processed using Infervision software (Infervision Medical Technology, Beijing, China) for 3D reconstruction and surgical planning (Fig. 1). The lesion was located inferior to the pulmonary artery and posterior to the junction of the left superior pulmonary vein and left atrium. This anatomical relationship suggested that posterior dissection of the left superior pulmonary vein would facilitate mobilization of the anterior bronchial wall and facilitate sleeve anastomosis.

**Fig. 2:**
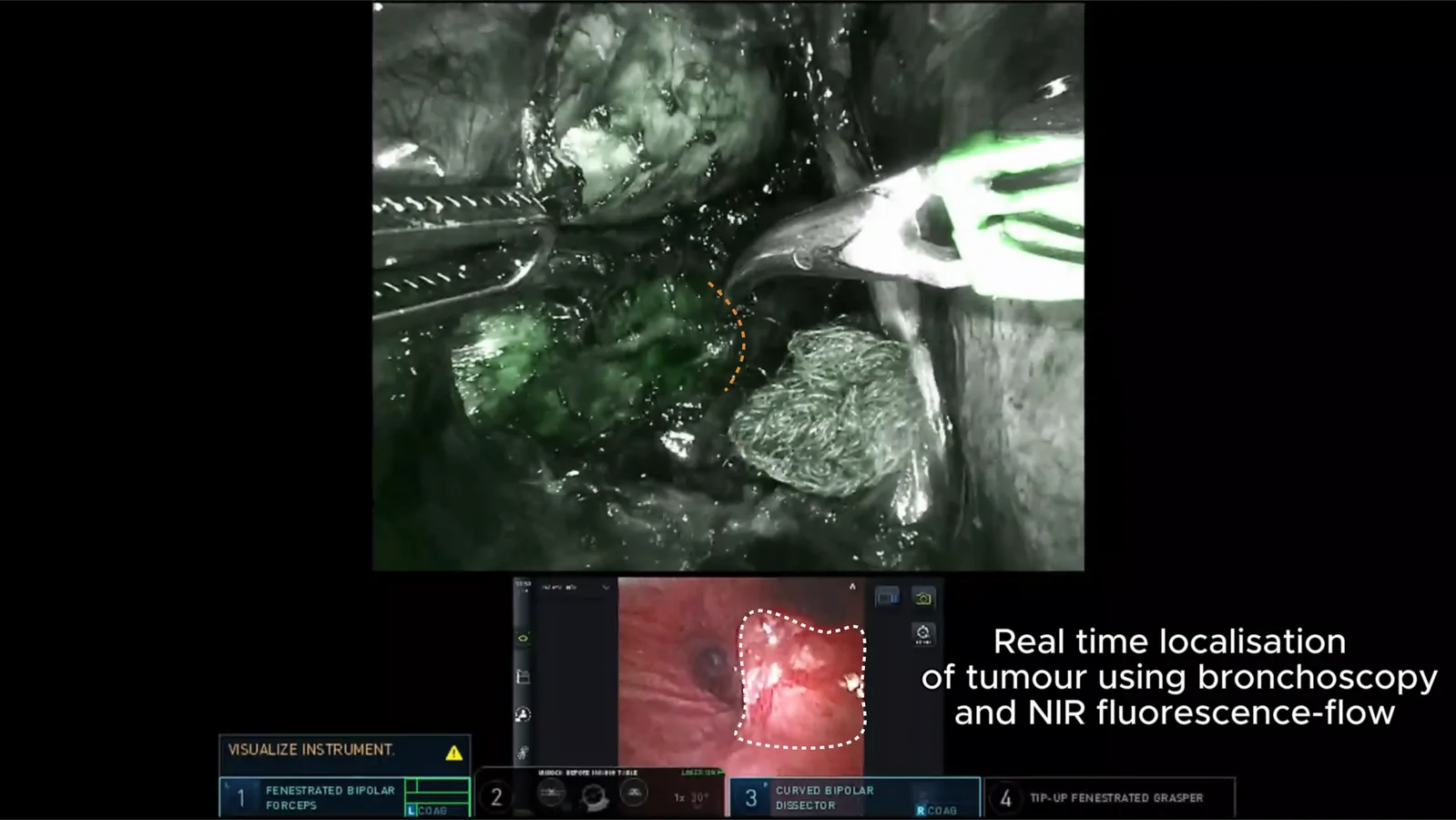
Real-time intraoperative tumor localization using Firefly near-infrared (NIR) fluorescence and concurrent bronchoscopy. The main panel demonstrates NIR green fluorescence signal transmitted through the bronchial wall, delineating the tumour margin to guide the proximal resection incision (orange dashed line). The inset panel shows the simultaneous bronchoscopic view of the endobronchial lesion (white dashed border)

Intraoperatively, repeat flexible bronchoscopy confirmed the lesion at the distal left main bronchus. The patient was then positioned in the right lateral decubitus position, and robotic ports were placed in a standardized configuration using three 8 mm and two 12 mm incisions, including a 12 mm AirSeal port (ConMed, Utica, NY) [5]. Robotic instruments using the da Vinci Xi (Intuitive Surgical, Sunnyvale, CA) platform included fenestrated bipolar forceps, spatula, curved dissector, fenestrated tip-up forceps, and staplers. Systematic nodal dissection was performed, including subcarinal and station 10 lymph nodes between the bronchus and pulmonary artery, which facilitated mobilization of the left main bronchus. Additional anterior mediastinal dissection and mobilization of the left superior pulmonary vein further exposed the anterior bronchial plane. Firefly near-infrared fluorescence imaging, used concurrently with flexible bronchoscopy, enabled real-time localization of the tumor through the bronchial wall and guided the proximal resection incision without the need for direct bronchial instrumentation or needle puncture (Fig. 2) [6].

**Fig. 3:**
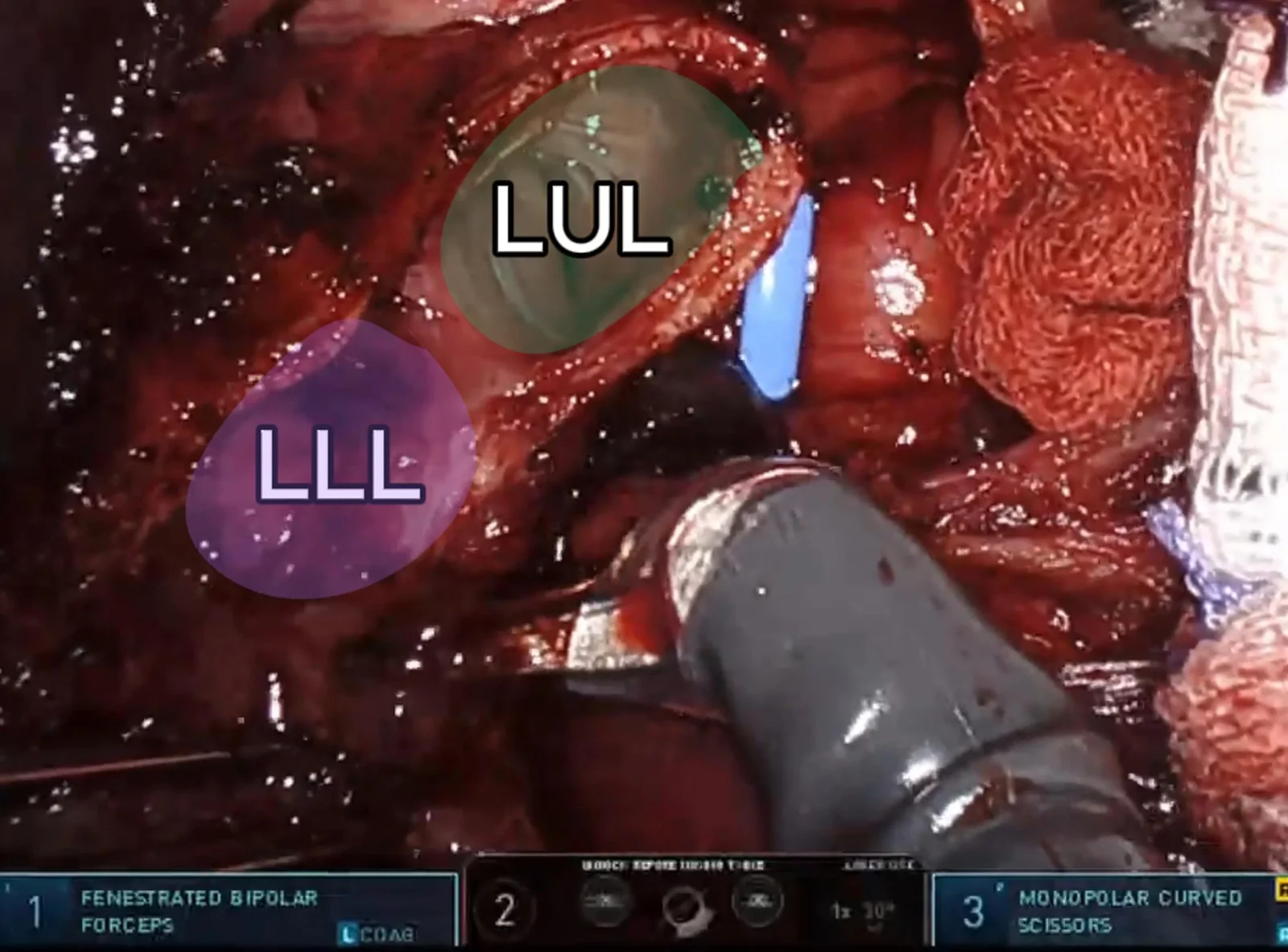

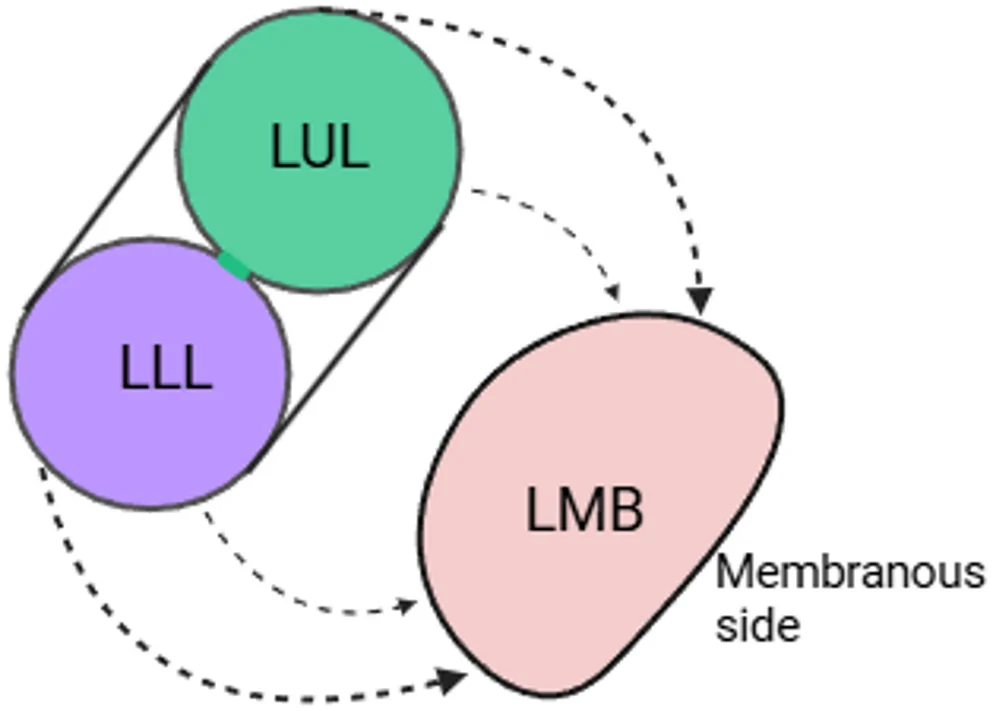
Left upper/lower lobe bronchi of the “figure 8” distal configuration. Figure 3b Anastomotic configuration; the curved section of the “D” proximal stump is cartilaginous, while the straight section is membranous. Dashed lines convey suture pattern. LUL, left upper lobe; LLL, left lower lobe; LMB, left main bronchus

**Fig. 4:**
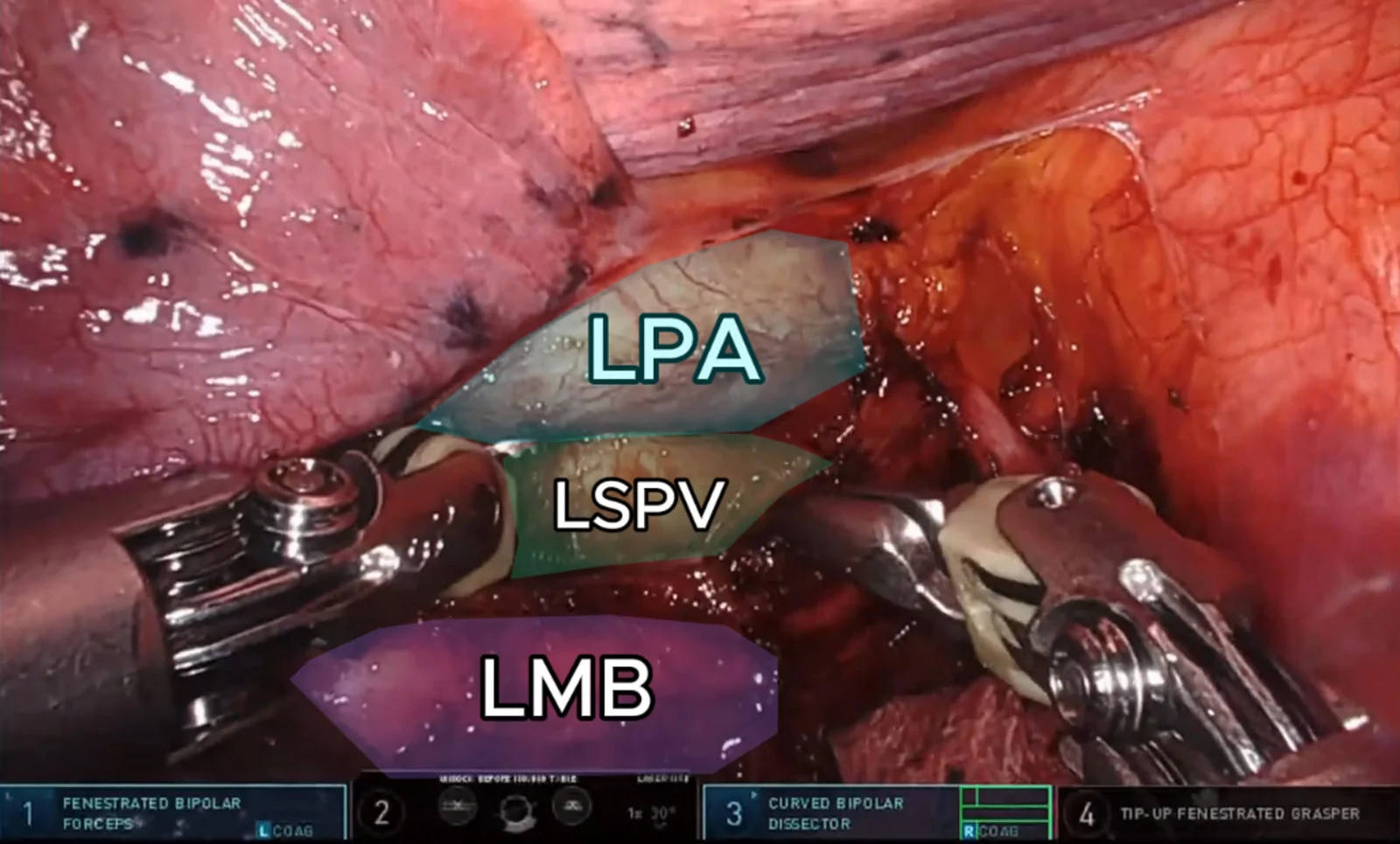
Adjacent vascular structures to the left main bronchus (LMB). LPA, left pulmonary artery; LSPV, left superior pulmonary vein

Circumferential bronchial division was performed using robotic scissors, allowing clear visualization of tumour margins. Distal resection was undertaken with care to achieve oncologically adequate margins. The specimen was submitted for intraoperative frozen section, confirming margin negativity. The bronchial ends were fashioned to create an “8”-shaped distal configuration incorporating the left upper and lower lobe bronchi, and a “D”-shaped proximal left main bronchus (Fig. 3a and b). Bronchial anastomosis was performed using a 3 − 0 Stratafix barbed suture (Ethicon, Somerville, NJ), commencing at the cartilaginous portion to optimize suture anchorage. Meticulous care was taken to avoid injury to adjacent vascular structures, including the pulmonary artery, pulmonary veins, and left atrium (Fig. 4). After completion of the posterior wall, a second suture was used to approximate the membranous portion. The sutures were then tightened and secured robotically. An underwater leak test confirmed anastomotic integrity, and a pedicled tissue flap was placed over the anastomosis to support healing. The patient had an uneventful recovery and was discharged after one week following physiotherapy and mobilization. Histopathology confirmed clear surgical margins without nodal involvement.

## Discussion

This case illustrates a minimally invasive, lung-sparing approach for resection of an endobronchial carcinoid tumour of the left main bronchus, avoiding pneumonectomy and thoracotomy. Two technical considerations merit emphasis. Although lesions of the left main bronchus can be approached via a right thoracic approach, the distal position of the lesion in this case favored a left transthoracic approach. Additionally, distal bronchial resection should preserve the bronchial wall between the left upper and lower lobe bronchi. This allows the distal “8” shaped airway configuration to be anastomosed to the proximal “D” shaped left main bronchus, rather than anastomosing two separate circular bronchi to the proximal segment, a technique that may increase the risk of air leak. The robotic platform offers specific advantages for this indication: the depth and confined anatomy of the left hilum are well-suited to the enhanced 3D visualization and mobility of the da Vinci Xi system, facilitating precise bronchial suturing and caliber adjustment in a field that is technically challenging by conventional thoracoscopy or open thoracotomy. The deep mediastinal position of the left main bronchus, its close relationship to the pulmonary artery, superior pulmonary vein, and left atrium, and the need for meticulous anastomotic technique each benefit from the degrees of freedom afforded by robotic instrumentation. Limitations of this approach include the institutional learning curve for complex robotic bronchoplastic operations, and the limited accessibility of the robotic platform in some centers. While prior reports have described robotic left main bronchial sleeve resection [6–8], the contribution of the present report lies in the integration of detailed preoperative 3D anatomical planning to guide dissection around critical proximal structures, and the description of the “8”-to-“D” bronchial reconstruction technique to address the geometric discrepancy between the distal and proximal airway configurations.

## Supplementary Information

Below is the link to the electronic supplementary material.

Supplementary Material 1
